# Feasibility of circulating tumor DNA analysis in dogs with naturally occurring malignant and benign splenic lesions

**DOI:** 10.1038/s41598-022-09716-6

**Published:** 2022-04-15

**Authors:** Patricia Filippsen Favaro, Samuel D. Stewart, Bradon R. McDonald, Jacob Cawley, Tania Contente-Cuomo, Shukmei Wong, William P. D. Hendricks, Jeffrey M. Trent, Chand Khanna, Muhammed Murtaza

**Affiliations:** 1 Translational Genomics Research Institute (TGen), Phoenix, AZ USA; 2 Department of Surgery and Center for Human Genomics and Precision Medicine, University of Wisconsin-Madison, Madison, WI USA; 3 Ethos Veterinary Health, Woburn, MA USA; 4 Ethos Discovery, San Diego, CA USA

**Keywords:** Cancer genomics, Biomarkers, Tumour biomarkers

## Abstract

Comparative studies of naturally occurring canine cancers have provided new insight into many areas of cancer research. Development and validation of circulating tumor DNA (ctDNA) analysis in pet dogs can help address diagnostic needs in veterinary as well as human oncology. Dogs have high incidence of naturally occurring spontaneous cancers, demonstrate molecular heterogeneity and clonal evolution during therapy, allow serial sampling of blood from the same individuals during the course of disease progression, and have relatively compressed intervals for disease progression amenable to longitudinal studies. Here, we present a feasibility study of ctDNA analysis performed in 48 dogs including healthy dogs and dogs with either benign splenic lesions or malignant splenic tumors (hemangiosarcoma) using shallow whole genome sequencing (sWGS) of cell-free DNA. To enable detection and quantification of ctDNA using sWGS, we adapted two informatic approaches and compared their performance for the canine genome. At the time of initial clinical presentation, mean ctDNA fraction in dogs with malignant splenic tumors was 11.2%, significantly higher than dogs with benign lesions (3.2%; *p* = 0.001). ctDNA fraction was 14.3% and 9.0% in dogs with metastatic and localized disease, respectively (*p* = 0.227). In dogs treated with surgical resection of malignant tumors, mean ctDNA fraction decreased from 11.0% prior to resection to 7.9% post-resection (*p* = 0.047 for comparison of paired samples). Our results demonstrate that ctDNA analysis is feasible in dogs with hemangiosarcoma using a cost-effective approach such as sWGS. Additional studies are needed to validate these findings, and determine the role of ctDNA to assess burden of disease and treatment response in dogs with cancer.

## Introduction

Comparative studies of naturally occurring canine cancers provide a unique opportunity to investigate unanswered questions in cancer biology, genomics, diagnosis, and therapy^1–3^. It is estimated that one in four dogs develop naturally occurring cancers during their lifetime. These cancers are generally diagnosed at late metastatic stages when symptomatic dogs present to veterinary clinics, often with complications. Diagnoses are confirmed with tissue biopsies, and dogs with cancer are treated with some combination of surgical resection, chemotherapy and targeted therapy^4^. Histopathological and molecular analyses of canine cancers have revealed many similarities with human cancer^5^. Somatic genomic alterations in canine cancer often affect driver genes well known for their role in human cancer, as observed in canine splenic hemangiosarcoma^6^. Analysis of somatic mutations in canine cancers can also yield novel insights into pathogenesis of specific cancer types^7^. Unlike murine preclinical models, the molecular heterogeneity seen in spontaneous cancers in dogs mimics human cancer heterogeneity, enabling the potential study of clonal evolution and acquired therapeutic resistance^8,9^.

There are few established circulating biomarkers for dogs with cancer. Analysis of circulating tumor DNA (ctDNA) in dogs can help address this gap for therapeutic development and routine veterinary care. It may help further our understanding of ctDNA biology, and enable development and refinement of novel diagnostic applications. Compared to preclinical models such as mice or rats, developing ctDNA analysis in dogs diagnosed with naturally occurring cancers presents several advantages including: (1) high incidence of spontaneous cancer in dogs (some of which are rare in humans such as sarcomas); (2) opportunity to collect serial blood samples of sufficient volume; (3) potential to collect concurrent samples of tumor tissue through repeatable biopsies; (4) routine use of clinical annotation methods in veterinary oncology that are analogous to those commonly used in human oncology (such as imaging, tumor grade, and patient stage); and (5) comparable relative sizes of tumors between dogs and humans. There are also several potential applications of ctDNA analysis in veterinary oncology^9^ including earlier detection of cancer^10^, noninvasive genotyping for actionable mutations^7^, real-time analysis of genomic evolution^11^, monitoring of treatment response^12^, monitoring for development of therapeutic resistance^13^, and detection of residual disease^14^. However, published experience with ctDNA analysis in dogs is currently limited. Earlier studies have suggested total cell-free DNA concentrations are higher in dogs with malignant tumors compared to those with benign disease or healthy controls^15–17^. In a recent study of canine mammary carcinoma, ctDNA detection was reported using digital PCR assays specific to somatic genomic rearrangements in 4 dogs^18^. In another study, ctDNA detection was demonstrated in 8 of 11 dogs with urothelial carcinoma using a real-time PCR assay for a recurrent somatic mutation in BRAF^15^. Earlier, we showed detection of ctDNA in 2 of 6 dogs with pulmonary adenocarcinoma using a digital PCR assay for a recurrent somatic mutation in ERBB2^7^. Hence, published reports of ctDNA analysis are limited to a few dogs and have largely used mutation and locus-specific assays that either rely on highly recurrent mutations affecting known cancer genes or require prior analysis of the tumor sample to identify patient-specific mutations. In this study, we have evaluated an alternative approach through the use of shallow Whole Genome Sequencing (sWGS) for ctDNA analysis of canine cancer, which relies on direct genome-wide assessment of copy number aberrations in plasma DNA.

Splenic hemangiosarcoma (HSA) is a relatively common canine cancer with strong clinical and genomic similarities to human angiosarcoma^19^. Both cancers harbor structurally complex genomes that are not associated with recurrent point mutations, limiting the utility of targeted resequencing gene panels. Hemangiosarcoma commonly develops in the spleen of dogs and metastasizes to distant organs prior to or early after initial diagnosis. These splenic lesions are not often discovered until they rupture and the dog presents to the veterinary hospital with acute abdominal hemorrhage, requiring emergent surgery. This clinical presentation is very similar to dogs with benign tumors of the spleen or other causes of splenic rupture. Histopathological confirmation of the diagnosis for these lesions cannot usually be achieved until several days after emergency surgeries are performed, highlighting the challenges that pet owners face when choosing to pursue aggressive emergent surgical care and treatment for their dog with an unclear long-term prognosis. An opportunity exists to study and translate methods for ctDNA analysis in this naturally occurring cancer model, to evaluate whether ctDNA levels can distinguish benign from malignant tumors, if ctDNA levels are related to burden of disease at presentation (such as localized vs. metastatic cancer), and if serial changes in ctDNA levels are effective surrogates for assessment of treatment response or disease progression. Here, we present a feasibility study evaluating the potential for ctDNA analysis using sWGS in dogs presenting with benign or malignant splenic tumors. We hope our findings and adapted informatics tools will help expand the inclusion of dogs with naturally occurring cancers in diagnostic and drug development efforts for veterinary and human oncology.

## Results

### Cell-free DNA analysis in healthy dogs

**Figure 1:**
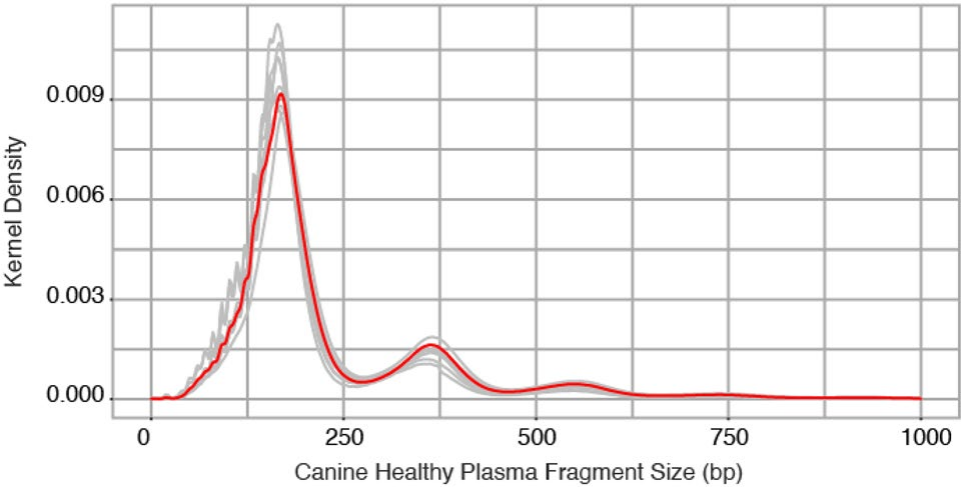
Plasma cell-free DNA fragment size (base pairs) in nine healthy dogs. Individual samples are shown in grey, all samples combined are shown in red.

To establish the feasibility of cell free DNA (cfDNA) analysis in dogs, we obtained plasma samples from 9 healthy dogs including five females and four males. The mean cfDNA concentration was 0.39 ng/ml of plasma (sd 0.38 ng/ml). We prepared sequencing libraries using a mean of 0.6 ng of plasma DNA (sd 0.2 ng) and generated a mean of 20.0 million sequencing read pairs per sample, achieving a mean unique sequencing depth of 0.45 × per sample (sd 0.15x). Following alignment to the dog genome CanFam3.1, we observed an average modal fragment size of 165.6 base pairs (bp) across 9 samples, comparable to the known modal fragment size of 166 bp in human cfDNA, which is associated with the length of DNA wrapped in mono-nucleosomes^20,21^ (Fig. 1, Table S1). Furthermore, the profile of cfDNA in healthy dogs showed similar 10 bp periodicity, and additional modes at fragment sizes associated with di- and tri-nucleosomes^22^.

### Clinical cohort of dogs with splenic lesions

We analyzed samples from a prospective clinical trial of dogs presenting with hemoabdomen secondary to presumed splenic rupture. A description of this clinical cohort of dogs, including diagnoses and perioperative outcomes, has been recently reported^23^. Samples from 39 dogs from this cohort were available for cfDNA analysis. Twenty five dogs (64.1%) were classified as having malignant splenic neoplasms and 14 (35.9%) were diagnosed with benign splenic lesions. Among the 25 dogs that were diagnosed with malignant tumors, 23 (92%) were diagnosed with HSA and 2 were diagnosed with stromal sarcoma. Two (2/25) of the dogs within the malignant tumor cohort had concomitant benign lesions: (1/2) splenic complex hyperplasia with hematoma, and (1/2) urinary bladder leiomyoma. Among the dogs with malignant tumors that had at least three blood samples collected (21/25), 9 had metastasis diagnosed at time of surgery and 7 were diagnosed with metastasis 36–203 days after surgery. Benign complex nodular hyperplasia represented 79% (11/14) of the benign lesions, while complex hyperplasia with hematoma, hematoma alone and myelolipoma represented 7% (1/14) each.

### Comparison of informatic approaches for sWGS

**Figure 2:**
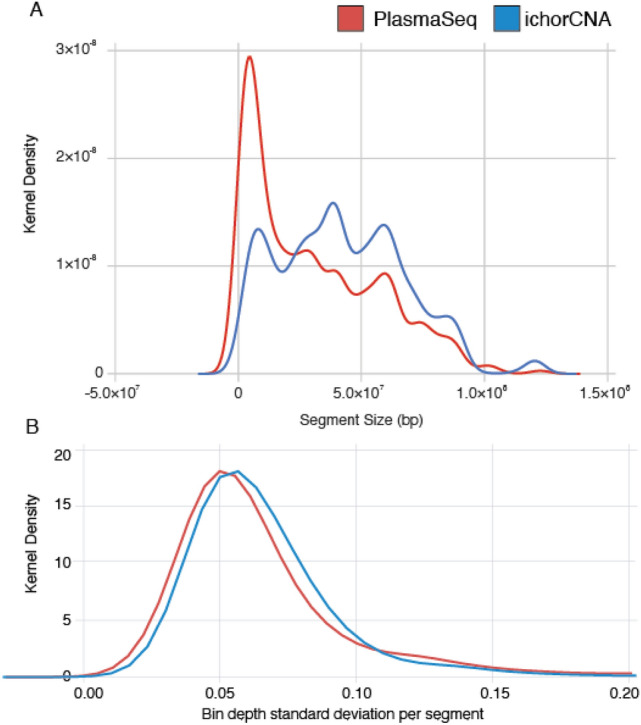
PlasmaSeq infers a larger proportion of shorter segments compared to ichorCNA, although the depth variation of bins within segments is approximately the same between PlasmaSeq and ichorCNA. (**A**) Distribution of segment size in base pairs across all samples using each approach. (**B**) The distribution of standard deviations of log_2_ depth across all bins assigned to the same segment.

To analyze sWGS data from cell-free DNA in dogs, we adapted two published informatic approaches, ichorCNA^24^ and PlasmaSeq^25^, and enabled their application to non-human genomes. Both tools infer the presence of tumor-derived copy number alterations (CNAs) using read depth in large, non-overlapping genomic bins (windows). Consecutive bins with the same copy number status are then grouped into segments. ichorCNA uses fixed-size bins while PlasmaSeq uses a fixed total number of bins and the boundaries are adjusted so that each bin contains approximately the same number of mappable bases. ichorCNA uses the magnitude represented by the detected CNAs to directly infer the fraction of tumor DNA found in blood. Canine plasma samples were analyzed using 500 kb bins for ichorCNA. PlasmaSeq analysis was run using 5500 total bins, resulting in a median size of 392 kb (sd 42 kb). Across all plasma samples from dogs with splenic lesions, the total number of segments was significantly higher for PlasmaSeq, with an average of 72.6 segments per sample versus 50.0 segments per sample for ichorCNA. Consistent with that observation, a larger fraction of copy number segments identified by PlasmaSeq were shorter compared to ichorCNA (Fig. 2A). To determine how precise each segment’s copy number assignment was by each tool, we quantified the amount of variation in read depth (log_2_ values) within bins assigned to a given copy number segment. The median standard deviation within each segment for ichorCNA and PlasmaSeq were 0.0606 and 0.0569 respectively, (Fig. 2B, *p* = 0.004 Mann–Whitney U). Although this suggests PlasmaSeq copy number assignments were more precise, in practice, these differences were marginal and we found the two adapted approaches to be equally useful for cfDNA analysis in dogs.

### Analysis of total cell-free DNA in dogs with cancer

At presentation of a dog, prior to any clinical or surgical intervention, mean concentrations of total cfDNA in plasma samples were 10.6 ng/ml and 20.3 ng/ml in dogs with benign and malignant lesions, respectively (*p* = 0.400). Within dogs with malignant lesions, mean total cfDNA concentration was 20.9 ng/ml and 19.5 ng/ml in dogs with localized and metastatic disease, respectively (*p* = 0.830). After surgery, total cfDNA concentration in plasma was significantly lower than baseline in both, dogs with benign (*p* = 0.008) and malignant disease (*p* = 0.017) (Table S2).

### Analysis of circulating tumor DNA in dogs with cancer

**Figure 3:**
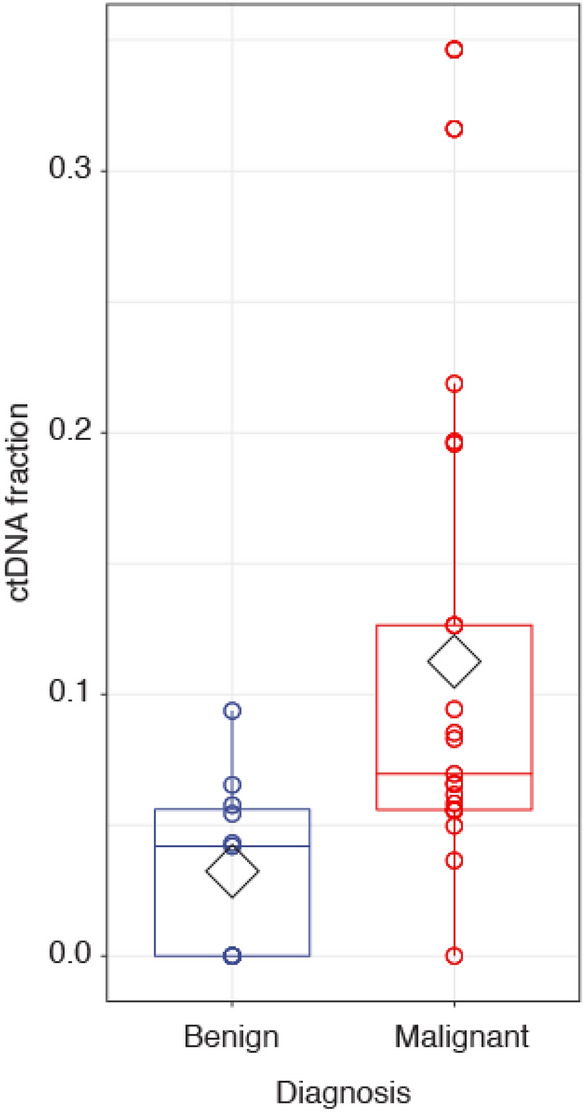
Circulating tumor DNA fraction for benign lesions vs malignant tumors in pre-treatment plasma samples (*p* = 0.001). Means are represented by diamonds for each group.

Whole genome sequencing libraries were prepared using a mean of 7.8 ng of input DNA (sd 11.1 ng). Sequencing was performed to generate a mean of 17.3 million read pairs (sd 8.2) achieving a mean sequencing coverage of 0.79× (sd 0.38×). Circulating tumor DNA fractions reported here were measured using an adapted version of ichorCNA. At presentation of a dog and prior to surgery, mean ctDNA fraction was 3.2% (sd 3.4) and 11.2% (sd 9.1) in dogs with benign lesions and malignant tumors, respectively (*p* = 0.001; Fig. 3, Table S2). ctDNA was detected above the previously reported limit of detection for ichorCNA of 3% tumor fraction in 20/21 dogs with malignant tumors with a pre-surgery sample available (95.2%). Based on measured ctDNA fractions, we were able to distinguish blood samples from dogs with benign lesions and malignant tumors with an area under the ROC curve of 0.84. Within dogs with malignant tumors, mean ctDNA fraction in dogs with localized and metastatic disease were 9.0% and 14.3%, respectively (*p* = 0.227). Following splenectomy, patients with malignant tumors showed a significant decrease in ctDNA levels from 11.0 to 7.9% (paired *p* = 0.047; Table S2). Interestingly, when corresponding tumor biopsies were analyzed using sWGS, we observed mean tumor fractions of 4.4% (sd 4.9) and 10.1% (sd 12.9) for benign lesions and malignant tumors, respectively, highlighting the challenges of obtaining high-cellularity splenic tumor biopsies.

**Figure 4:**
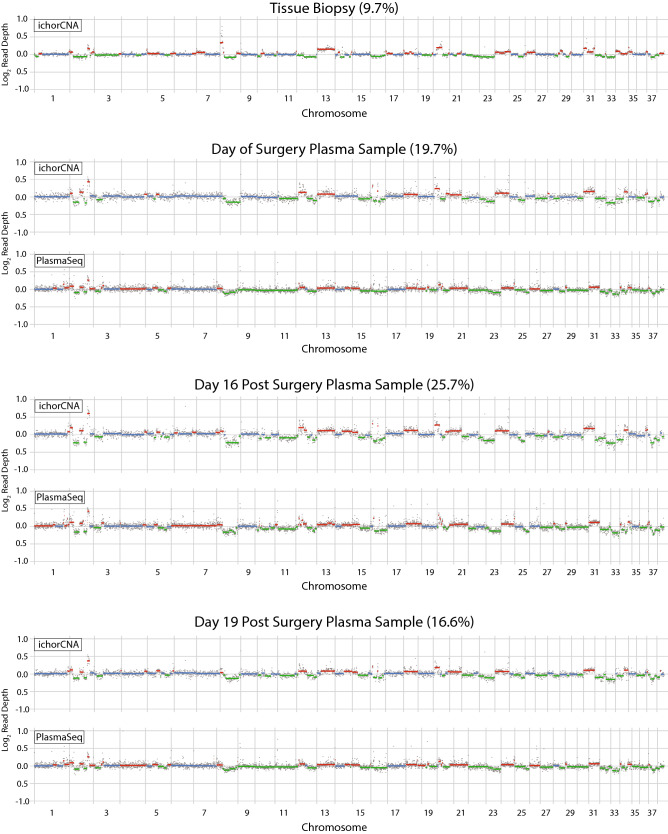
Genome-wide copy number variation plots from the metastatic HSA canine patient, dog no. 26, representing the plasma and tumor biopsy sWGS from day of surgery, and plasma sWGS from days 16 and 19, both after the splenectomy procedure.

**Figure 5:**
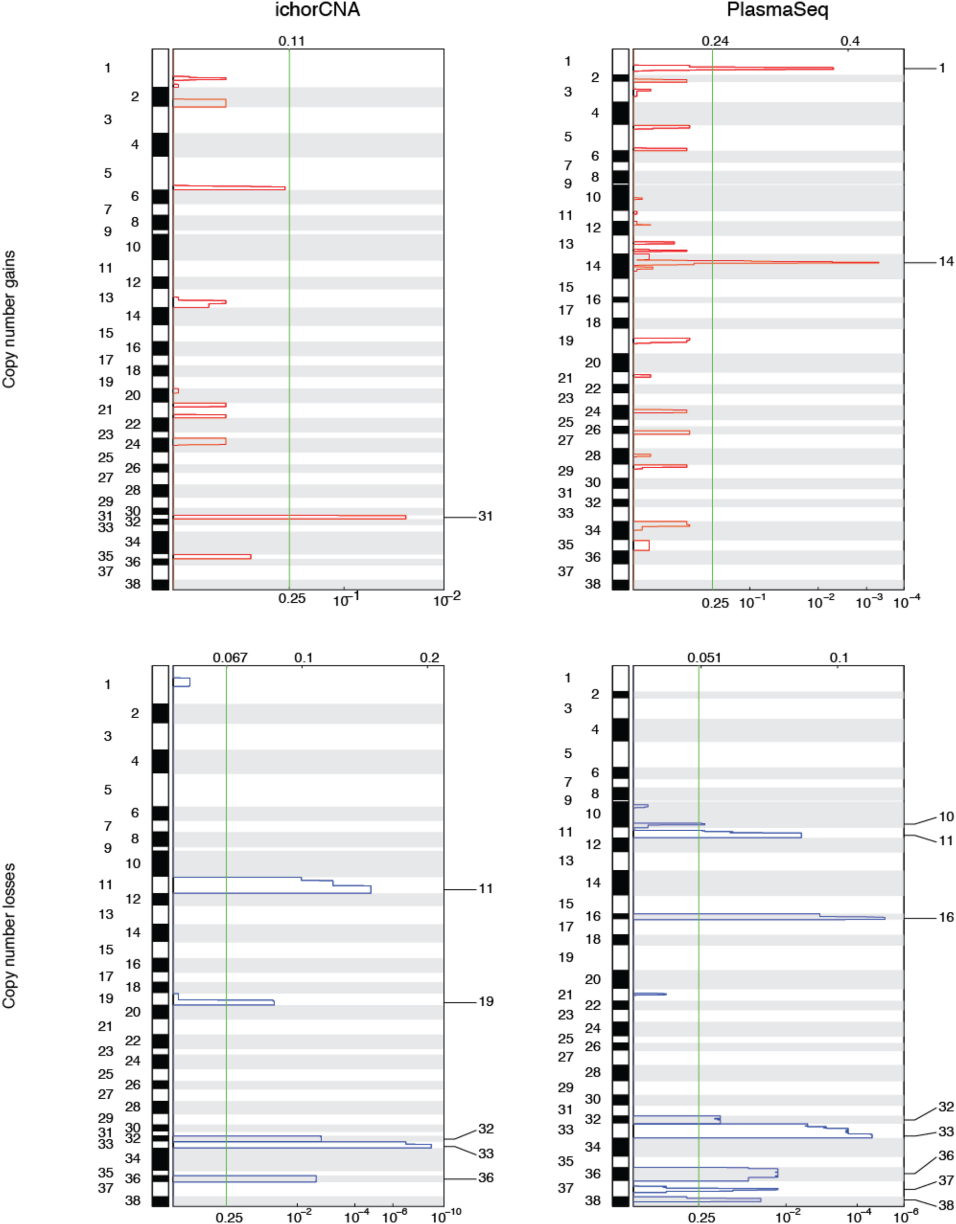
Assessment of significant recurrence for observed copy number gains (top) and copy number losses (bottom), using the two approaches, ichorCNA (left) and PlasmaSeq (right). Copy number deletions observed on chromosomes 11, 32, 33 and 36 were consistent across the two approaches.

In cases where corresponding tumor DNA and cfDNA samples both showed adequate tumor fraction, copy number aberrations observed were largely concordant. For example, in one dog with metastatic malignant hemangiosarcoma, copy number aberrations observed in tumor biopsy are also observed in blood samples obtained on days 0, 16 and 19 after splenectomy, using both informatic approaches (Fig. 4). To determine whether recurrent copy number alterations were observed across plasma samples in this cohort, we utilized a published algorithm called GISTIC to assess significance of segments identified by PlasmaSeq and ichorCNA^26^. For each dog with malignant disease, one plasma sample with the highest tumor fraction observed was included in this analysis. Recurrent copy number gains that passed the threshold for cohort-wide significance were not consistent between ichorCNA and PlasmaSeq. In contrast, recurrent copy number losses were observed on chromosomes 11, 32, 33 and 36 using both approaches (Fig. 5). CDKN2B resides within the deletion peak on chromosome 11 identified in our analysis. Deletion of CDKN2A/B is consistent with prior published results from human angiosarcoma and canine hemangiosarcoma^19^.

Follow-up blood samples from two dogs classified as having (a) benign complex nodular hyperplasia and (b) complex nodular hyperplasia with hematoma, had unexpectedly high tumor fractions in plasma (21.4% and 46.9%). However, these observations were made at 392 and 345 days after surgery, respectively. In both dogs, earlier samples had ctDNA levels consistent with those observed in dogs with benign disease (5.4% and 4.8%). Post hoc analysis revealed new neurologic clinical signs that were localized to the right cerebral cortex 415 days following surgery in one of the two dogs suggestive of distinct intracranial neoplasia. However, further investigation was not pursued to confirm this diagnosis. There were no clinical signs suggestive of neoplasia in the second dog.

## Discussion

ctDNA analysis in pet dogs with cancer provides an opportunity to address diagnostic gaps in veterinary oncology as well as to develop novel methods and applications for liquid biopsies in human oncology. Analysis of naturally-occurring cancers with similar complexities of cancer heterogeneity as human patients can help advance the use of liquid biopsies for tracking cancer evolution during treatment, potentially generating novel insights. However, published experience with ctDNA analysis in canine oncology is still limited. In the current study, we demonstrate that sWGS is feasible as a tumor-independent approach for ctDNA detection in dogs with cancer. We adapted informatic methods to analyze sWGS data for application to the canine genome and make these versions available for future use to accelerate the development and deployment of ctDNA analysis in dogs^24,25^. We report proof-of-principle results showing higher levels of ctDNA in dogs presenting with malignant splenic tumors compared to those with benign disease. We found that the overall fragment size distribution of plasma DNA in healthy dogs was similar to that observed in human plasma DNA previously, including a mode at 166 bp and 10 bp periodicity^27^. The analysis of ctDNA levels in serial samples collected before and after resection of the splenic tumors showed ctDNA levels were significantly lower after surgery. Comparison of dogs with localized versus metastatic disease (noted at the time of surgery) were not statistically distinct. This observation may be potentially explained by the identification of metastasis days or weeks (a range of 46 to 203 days) after surgery in 7 of 12 dogs with malignant tumors who appeared to have localized disease at presentation. Moreover, comparison of localized vs. metastatic disease may be affected by a limited sample size and the presence of micro-metastasis in some dogs that remained undetectable using routine veterinary diagnostic investigations (chest x-rays and abdominal ultrasounds).

Our results were obtained using shallow WGS achieving genome-wide sequencing coverage of less than 1 × on average by generating 15–20 million read pairs of DNA sequencing. Unlike deeper sequencing of focused cancer gene panels that may require several hundreds to thousands-fold sequencing coverage, sWGS has potential to be more cost-effective. To prepare sequencing libraries and generate data as reported in our current study, the cost of reagents will range from $50 to $100 per sample depending on the scale of the project and available laboratory resources (including choice of sequencing platform).

Although we found that ctDNA fraction was higher in dogs with malignant disease, most dogs with splenic tumors present in an emergency with ruptured spleen and hemoabdomen. The fastest turnaround time for the approach described here, even in the best case scenario with no logistical delays, is approximately 24 hours. Therefore, this approach is unlikely to aid emergent decision making as a point-of-care test. We also note that following resection of malignant splenic tumors, ctDNA fraction decreased but did not become undetectable as observed in earlier human studies^14^. Since malignant splenic tumors present late in dogs and have poor prognosis, it is possible that the high residual levels of ctDNA may be due to unidentified metastatic disease sites. If this is validated further in cohorts with more extensive annotation of clinical characteristics and survival, post-operative ctDNA analysis could help improve prognostication, inform the decision to start adjuvant therapy, and improve treatment monitoring in veterinary oncology.

This feasibility study has several limitations. ctDNA studies in human oncology have largely come to rely on gold standard pre-analytical processing focused on rapid separation of plasma samples. In on-going studies, we have ensured implementation of standard processing protocols to isolate plasma rapidly after venipuncture. In future work, ctDNA analysis in dogs may provide an opportunity to evaluate and optimize pre-analytical factors using paired tumor samples and multiple blood samples from cancer patients, such as blood collection tubes, DNA extraction methods, and storage conditions. An additional limitation was our detection of ctDNA in dogs with benign disease at presentation. As we note, ctDNA levels were significantly lower than dogs with malignant disease and most were observed at < 6% tumor fraction. These observations are close to the limit of detection previously reported for ichorCNA, which was extensively validated using human plasma DNA samples. In future studies, additional analytical validation is needed to ascertain the limit of detection from canine plasma DNA, and establish the accuracy of ctDNA detection at low tumor fraction. In addition, we observed significantly elevated levels of ctDNA detectable in two dogs with benign lesions in follow-up blood samples. The source of these elevated ctDNA levels observed later in follow-up is unclear. In these older clinical patients, it is possible that elevated ctDNA levels almost a year after initial diagnosis are a result of clinically silent unrelated cancers. These dogs were shown to have benign splenic lesions at the time of initial diagnosis and surgery but hematological or other malignancies present concomitantly or arising later may only become apparent during sufficient clinical follow-up. In one of these dogs, concomitant neoplasia of the brain was suspected, but not confirmed, based on the subsequent development of clinical signs suggestive of a space occupying mass. Given the clinical characteristics of this cohort of larger older dogs, we speculate that a non-splenic cancer, such as indolent lymphoma, may also explain some of these findings. In this study, we did not perform comparison of ctDNA levels with tumor volume on imaging. Future studies may include measurement of tumor volumes to account for quantitative differences in burden of metastatic disease and size of primary tumors, and how these may correlate with observed differences in ctDNA levels.

In summary, our results demonstrate that ctDNA analysis is feasible and holds potential for improving diagnostics in veterinary oncology. Future studies are warranted to further develop methods and applications of ctDNA analysis in larger cohorts of dogs with cancer. In addition, ctDNA analysis of naturally-occurring cancers in dogs can enable further optimization for diagnostic applications in human oncology including noninvasive tumor genotyping, assessment of disease burden, and monitoring of treatment response.

## Methods

### Ethics statement

Blood and tissue samples were collected with the approval and informed consent of the canine patient owners. The study was launched following approval from the animal care and use committee convened by Animal Clinical Investigation (Chevy Chase, MD). All methods were performed in accordance with relevant guidelines and regulations.

### Sample collection

Blood samples were collected from the jugular or cephalic vein from 39 canine patients who presented with acute hemoperitoneum secondary to splenic neoplasm rupture. All dogs underwent preoperative staging, the results of which have been previously reported^23^. The whole blood was transferred to Cell Free DNA BCT Streck tubes (Streck, Inc.), and processed as per manufacturer’s instructions to isolate plasma. Collected plasma and buffy coat were stored at – 80 °C. Blood sample collection was performed prior to splenectomy and at subsequent clinical visits. Through clinical and histopathological examinations, patients were diagnosed with malignant splenic neoplasms (n = 25) and benign splenic lesions (n = 14). All histopathologic diagnoses were verified through post hoc review of medical records and clinical data to confirm the diagnoses. Blood samples were also collected in Cell Free DNA BCT Streck tubes (Streck, Inc.) from nine healthy dogs and processed similarly.

### DNA extraction from tumor, white blood cells and plasma

Cell-free DNA was extracted from 1 to 4 ml of plasma using the MagMAX Cell-Free DNA Isolation Kit (Thermo Fisher Scientific, Austin, TX) or the QIAmp Circulating Nucleic Acid Kit (Qiagen, Hilden, Germany), as per manufacturers’ instructions. The tissue biopsy (30 mg) was rinsed in 1X phosphate buffered saline, homogenized with Bullet Blender Bead Lysis Kit (NextAdvance, Troy, NY) and supernatant was passed through QIAshredder columns (Qiagen, Hilden, Germany) before tumor DNA was extracted using the Allprep DNA/RNA/miRNA Universal Kit (Qiagen, Hilden, Germany), according to the manufacturer’s instructions. All extracted DNA were stored at – 20 °C until analysis. Plasma and tumor DNA concentration, integrity and purity were assessed using Bioanalyzer (Agilent Technologies, Santa Clara, CA), Qubit 2.0 Fluorometer (Thermo Fisher Scientific, Austin, TX) and 4200 TapeStation genomic DNA assay (Agilent Technologies, Santa Clara, CA). DNA from the white blood cells was extracted from 200 µl of buffy coat using the DNeasy Blood Tissue Kit (Qiagen, Hilden, Germany), according to the manufacturer’s instructions, and stored at – 20 °C until further analysis.

### Whole genome sequencing of plasma cfDNA and tumor DNA

Sequencing libraries from cfDNA were prepared with SMARTer ThruPLEX Plasma-Seq Kit and DNA HT Dual Index Kit (Takara Bio USA, Mountain View, CA), as per manufacturer’s instructions allowing up to 30 ng of input DNA. Sequencing libraries were purified with SPRI magnetic beads (Beckman Coulter, Brea, CA). Library sizes and concentrations were measured using a genomic DNA assay on the TapeStation 4200 (Agilent Technologies, Santa Clara, CA). Tumor DNA was fragmented to a target size of 200 bp on E220 Focused-ultrasonicator (Covaris, Woburn, MA). 20 ng of sheared DNA was used for the library construction with ThruPLEX DNA Seq Kit and DNA HT Dual Index Kit (Takara Bio USA, Mountain View, CA). Plasma and tumor DNA libraries were sequenced using paired-end 50 bp reads generated on the NovaSeq 6000 Sequencing System (Illumina, San Diego, CA).

### Somatic copy number analysis

Raw sequence data was converted to fastq using bcl2fastq v2.20.0.422. Reads were trimmed using fastp v0.2^28^ and then aligned to the canFam 3.1^29^ genome assembly using bwa-mem. WGS bam files from nine healthy canine plasma cfDNA samples were used as controls for ichorCNA and PlasmaSeq analysis of large-scale copy number changes. GenMap^30^ was used to calculate mappability on the canFam 3.1 genome assembly. PlasmaSeq analysis was conducted using our implementation of the algorithm using the Julia programming language v1.1. Usable bases were identified using the mappability data, and boundaries for the fixed 5500 bins were selected to reduce the variability of usable bases across all bins. The number of bins was selected to be approximately the same as the number of bins used by ichorCNA. For relative bin depth normalization, bams from the nine healthy control samples were merged. Read depths for PlasmaSeq bins were calculated using bedtools v2.28.0^31^.

ichorCNA analysis was conducted using a modified version of ichorCNA v0.3.2. Functions that standardized chromosome names for human genomes were removed, as they caused errors with unexpected chromosome names from the canine genome. A canFam 3.1 panel of normals was generated using the nine healthy control samples with 500 kb bins. Mappability and GC content calculations were performed as for PlasmaSeq. HMMcopy^32^ was used to calculate read depths per bin. Initial normal proportion range was set to [0.7, 0.8, 0.9, 0.95, 0.99]. Ploidy was fixed at 2, with a max copy number of 3 and subclone state options of [1,3]. Without these restricted parameter ranges, high tumor fraction inferences with no obvious copy number changes and inferred ploidy of 3 were very common across samples.

### Statistical analysis

Patient groups were compared using non-parametric tests including Kruskal–Wallis and Wilcoxon Rank-Sum tests using the R package *stats,* and plots were prepared using the packages *ggpubr*, *magrittr*, *ggplot2*, *ggsci* and *scales*. Specificity, sensitivity and test performance were calculated with the packages *ROCR* and *cvAUC*. All means, standard deviations and confidence intervals were calculated with the R package *stats*.

### Data access

The sequencing data generated in this study has been submitted to the Sequence Read Archive (SRA) under accession number PRJNA823593. Adapted versions of ichorCNA and PlasmaSeq have been deposited to github at https://github.com/ctdnalab/ichorCNA_U and https://github.com/ctdnalab/PlasmaCNA, respectively.

## Supplementary Information

Supplementary Information.
