# Supplementary material for: Feasibility of circulating tumor DNA analysis in dogs with naturally occurring malignant and benign splenic lesions
Source: Sci Rep. 2022 Apr 15;12:6337. doi: 10.1038/s41598-022-09716-6 (PMC9012871; doi:10.1038/s41598-022-09716-6)
Supplement: Supplementary file 1 — Supplementary Information. [file 41598_2022_9716_MOESM1_ESM.pdf]

Supplementary Materials for
Feasibility of circulating tumor DNA analysis in dogs with naturally-occurring malignant and benign splenic lesions

Patricia Filippsen Favaro, Samuel D. Stewart, Bradon R. McDonald, Jacob Cawley, Tania Contente-Cuomo, Shukmei Wong, William P.D. Hendricks, Jeffrey M. Trent, Chand Khanna*, Muhammed Murtaza*

*Co-corresponding authors:
Muhammed Murtaza (murtaza@surgery.wisc.edu)
Chand Khanna (ckhanna@ethosvet.com)

Healthy dog	cfDNA concentration (pg per ml of plasma)	Modal cfDNA fragment size (base pairs)	cfDNA WGS coverage
1	247.5	163	0.46
2	375.5	166	0.83
3	238.0	171	0.39
4	143.9	164	0.48
5	260.6	167	0.45
6	1448.9	165	0.30
7	310.7	153	0.33
8	151.2	171	0.45
9	349.5	170	0.42

Table S1. Description of cell free DNA concentration per mL of plasma and the respective modal fragment size and whole genome sequencing (WGS) coverage from the plasma of nine healthy dogs.

Patient	Gender	Breed	Age (years)	BW (kg)	Diagnosis	Survival (dps)	Pre-operative cfDNA conc.	First post-operative cfDNA conc.	Pre-operative ctDNA fraction	First post-operative ctDNA fraction	TF tumor biopsy	MT
2	Ms	American Pit Bull	6	40.4	Benign A	192**	4.38	0.19	0.094	0.063	0	No
3	Ms	Mix	10	25.1	Benign A	3	1.06	na	0	na	0	No
5	Ms	Mix	12	35	Benign A	369**	2.20	0.47	0	0.092	0	No
6	Fs	Labrador Retriever	5.83	30.9	Benign A	376**	1.91	0.15	0	0.049	0	No
7	Ms	Pointer mix	9	39.1	Benign A	740**	5.02	na	0.043	na	0.11	No
8	Fs	American Pit Bull	7	38.5	Benign A	388**	0.39	0.02	0.065	0.031	0.10	No
9	Ms	Wirehaired Point Griffon	10.92	33	Benign A	882**	64.47	na	0.058	na	0.075	No
10	Fs	English Bulldog	10	25	Benign A	581	0.28	0.27	0.043	0	0.074	No
11	Fs	Mix	11	13	Benign A	363**	35.85	0.33	0.054	0.054	0	No
12	Ms	Jack Russel Terrier	13	10	Benign C	389**	0.54	0.49	0	0	0.10	No
13	Fs	Corgi	14	11.3	Sarcoma	382	15.75	0.72	0.070	0.28	0.33	No
15	Fs	Labrador Retriever	11	63.4	Benign B	267	0.17	na	0	na	0	No
16	Ms	Standard Poodle	14	24.9	HSA I	50	0.71	0.35	0.066	0.10	0.28	No
17	Fs	Wheaten Terrier	11	20.6	HSA I	148	4.77	na	0.058	na	0.14	No
19	Fs	Labrador Retriever	11	28.2	HSA II	111*	0.22	1.04	0.13	0	0	Yes
20	Ms	Labradoodle	9	33.2	HSA II	15	10.47	na	0.13	na	0	No
21	Fs	Labrador Retriever	10	24.3	HSA II	2	4.26	0.09	0.056	0	0.078	No
22	M	Goldendoodle	12	55.6	HSA II	121	0.16	0.37	0.050	0.11	0.055	No
23	Fs	German Shepherd	11	36.2	HSA II	36	2.41	0.30	0.083	0	0	No
25	M	Great Dane	7.83	60	HSA II	77	1.23	0.21	0.055	0	0.13	Yes
26	Ms	Mix	11	32	HSA II	19	0.92	1.84	0.20	0.26	0.097	Yes
27	Ms	Boxer mix	6	37	HSA II	204	2.15	0.89	0.062	0	0	No
28	Fs	German Shepherd	12	37.8	HSA II	3	35.93	na	0.32	na	0.091	Yes
29	Fs	Boxer	7	28	HSA II	366**	175.80	1.28	0.056	0.044	0.16	No
30	Ms	English Setter	11	26	HSA III	0	0.08	na	0	na	0	Yes
33	Ms	Yorkshire Terrier Mix	8.25	6.8	HSA III	61	21.25	3.43	0.094	0.063	0.25	Yes
34	Fs	Mix	9	18.4	HSA III	40	0.60	2.97	0.35	0	0.20	Yes

35	Fs	Mix	11	21	HSA III	92	9.31	422.79	0.036	0.081	0.13	No
36	Fs	Mix	12	38	HSA III	195	0.85	0.09	0.22	0.25	0	No
37	Ms	Husky	10	33.5	HSA III	0	87.74	na	0.085	na	0	Yes
38	M	Golden Retriever	11	34.6	HSA III	18	28.43	0.02	0.066	0	N/A	Yes
39	Fs	Mix	12	22.3	Sarcoma	794	23.57	1.15	0.20	0	0	No

Table S2. Description of the patients included in the baseline peri-operative cohort, with the respective diagnostic, cell-free DNA concentrations and tumor fractions. M = male intact; Ms = male sterilized; Fs = female sterilized; dps = days post-surgery; na = not applicable; cfDNA conc = cell-free DNA concentration (ng) per mL of plasma; TF cfDNA = tumor fraction in plasma cell free DNA; TF tumor biopsy = ichor tumor fraction in tissue tumor biopsy; BW = bodyweight (Kg) at the time of surgery; MT = metastasis identified during peri-operative period; Benign A = Benign Complex Nodular Hyperplasia; Benign B= Myelolipoma; Benign C = Benign Complex Hyperplasia and Hematoma; * = last day seen; ** = alive at last day of study.
